# Mechanism of the Dual Activities of Human CYP17A1 and Binding to Anti-Prostate Cancer Drug Abiraterone Revealed by a Novel V366M Mutation Causing 17,20 Lyase Deficiency

**DOI:** 10.3390/ph11020037

**Published:** 2018-04-29

**Authors:** Mónica Fernández-Cancio, Núria Camats, Christa E. Flück, Adam Zalewski, Bernhard Dick, Brigitte M. Frey, Raquel Monné, Núria Torán, Laura Audí, Amit V. Pandey

**Affiliations:** 1 Growth and Development Research Unit, Vall d’Hebron Research Institute (VHIR), Center for Biomedical Research on Rare Diseases (CIBERER), Instituto de Salud Carlos III, Autonomous University of Barcelona, Barcelona 08035, Spain; monica.fernandez.cancio@vhir.org (M.F.-C.); nuria.camats@vhir.org (N.C.); laura.audi@vhir.org (L.A.); 2 Pediatric Endocrinology Unit, Department of Paediatrics, University Children’s Hospital Bern, Bern 3010, Switzerland; christa.flueck@dbmr.unibe.ch (C.E.F.); adam.zalewski@mail.com (A.Z.); 3 Department of Biomedical Research, University of Bern, Bern 3010, Switzerland; 4 Department of Nephrology and Hypertension, University of Bern, Bern 3010, Switzerland; Bernhard.dick@gmx.ch (B.D.); brigitte.frey@dbmr.unibe.ch (B.M.F.); 5 Pediatric Service, Hospital Joan XXIII, Tarragona 43005, Spain; raquel.monne@urv.cat; 6 Pathology Department, Hospital Universitari Vall d’Hebron, CIBERER, Barcelona 08035, Spain; toranfuentesn@gmail.com

**Keywords:** P450c17, prostate cancer, abiraterone, steroidogenesis, androgens, dehydroepiandrosterone, *CYP17A1*, cytochrome P450, anti-cancer drugs, DSD

## Abstract

The *CYP17A1* gene regulates sex steroid biosynthesis in humans through 17α-hydroxylase/17,20 lyase activities and is a target of anti-prostate cancer drug abiraterone. In a 46, XY patient with female external genitalia, together with a loss of function mutation S441P, we identified a novel missense mutation V366M at the catalytic center of CYP17A1 which preferentially impaired 17,20 lyase activity. Kinetic experiments with bacterially expressed proteins revealed that V366M mutant enzyme can bind and metabolize pregnenolone to 17OH-pregnenolone, but 17OH-pregnenolone binding and conversion to dehydroepiandrosterone (DHEA) was impaired, explaining the patient’s steroid profile. Abiraterone could not bind and inhibit the 17α-hydroxylase activity of the CYP17A1-V366M mutant. Molecular dynamics (MD) simulations showed that V366M creates a “one-way valve” and suggests a mechanism for dual activities of human CYP17A1 where, after the conversion of pregnenolone to 17OH-pregnenolone, the product exits the active site and re-enters for conversion to dehydroepiandrosterone. The V366M mutant also explained the effectiveness of the anti-prostate cancer drug abiraterone as a potent inhibitor of CYP17A1 by binding tightly at the active site in the WT enzyme. The V366M is the first human mutation to be described at the active site of CYP17A1 that causes isolated 17,20 lyase deficiency. Knowledge about the specificity of CYP17A1 activities is of importance for the development of treatments for polycystic ovary syndrome and inhibitors for prostate cancer therapy.

## 1. Introduction

**Figure 1:**
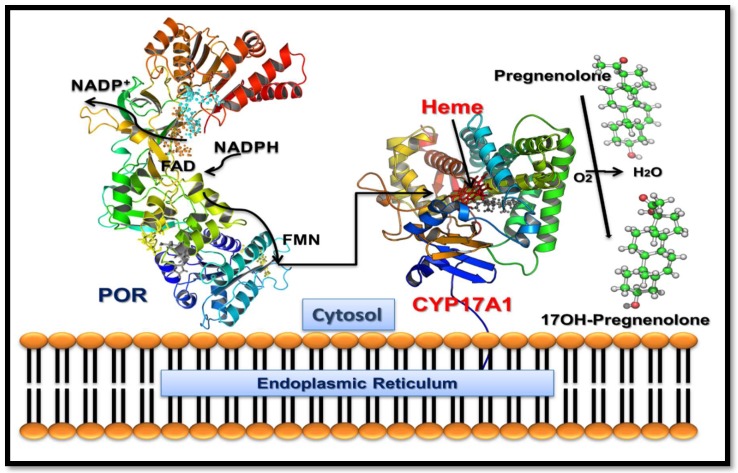
Schematic diagram of CYP17A1 and P450 oxidoreductase (POR) interaction in the membranes. The nicotinamide adenine dinucleotide phosphate (NADPH) molecules bind to POR, which is embedded into the membranes of endoplasmic reticulum and donate a pair of electrons, one at a time, which are received by the FAD. Transfer of electrons to FAD creates a change in conformation of POR, allowing the FAD and FMN groups to move towards each other, which facilitates the transfer of electrons from FAD to FMN. The FMN domain of POR interacts with the CYP17A1 and transfers electrons for catalytic activities.

**Figure 2:**
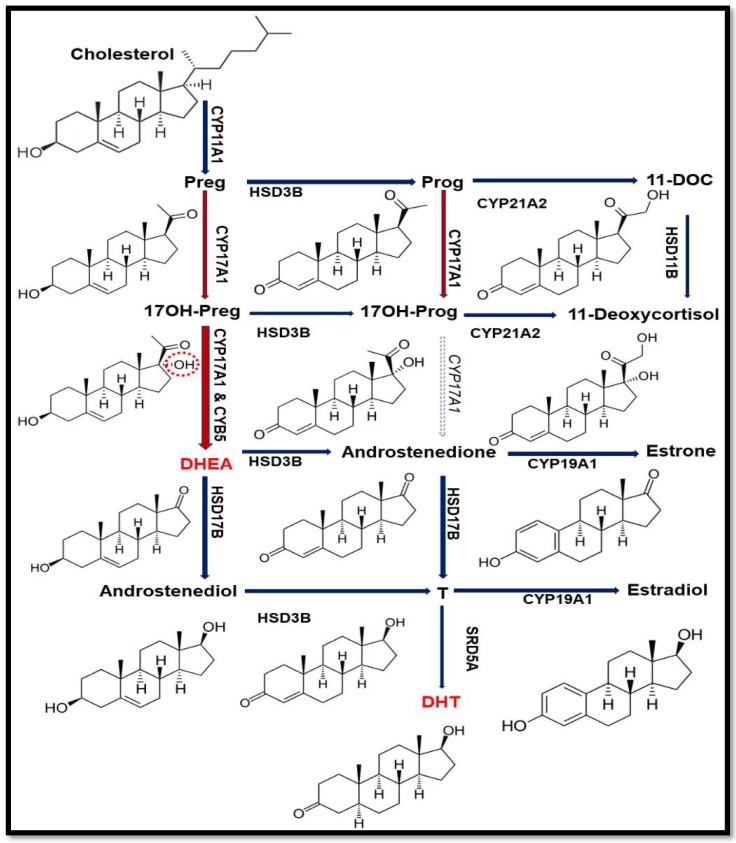
Steroid biosynthetic pathway. In humans, 17,20 lyase activity of *CYP17A1* converts 17α-hydroxypregnenolone (17OH-PREG) to dehydroepiandrosterone (DHEA) but does not effectively convert 17α-hydroxyprogesterone (17OH-PROG) to androstenedione. The DHEA is the precursor for androgen production and (dihydrotestosterone) DHT is the potent form of androgen with higher affinity towards androgen receptor (AR) than testosterone (T). The 17α-hydroxy position of 17OH-PREG is highlighted in red to show the difference from PREG.

The Cytochrome P450 proteins that are located in the endoplasmic reticulum are responsible for the metabolism of xenobiotics, drugs and steroid hormones (Figure 1) and are part of the microsomal mixed oxidase system [1,2,3]. Cytochrome P450c17 (CYP17A1) is required for the biosynthesis of steroid hormones in all vertebrates. The CYP17A1 is the qualitative regulator of the biosynthesis of sex steroid in humans (Figure 2) [4]. CYP17A1 catalyses multiple reactions in the steroid pathway [5,6,7]; chiefly among them, its 17α-hydroxylase activity is essential for the production of 17OH-pregnenolone (17OH-PREG) and 17OH-progesterone (17OH-PROG) which are precursors of cortisol, and its 17,20 lyase activity is required for the generation of the precursor of sex steroids, dehydroepiandrosterone (DHEA) (Figure 2). These two activities of the CYP17A1 determine the type of steroid hormone synthesized in different cells and tissues; if the CYP17A1 is absent, mineralocorticoids are produced, if only the 17α-hydroxylase activity of the CYP17A1 is present, glucocorticoids are made; and if both the 17α-hydroxylase and the 17,20 lyase activities of the CYP17A1 are present, sex steroid precursors are generated [4]. Overproduction of androgens by the specific activation of CYP17A1-17,20 lyase activity has been implicated in the pathogenesis of the polycystic ovary syndrome [4]. CYP17A1 is a target for prostate cancer therapy by inhibitor abiraterone (Zytiga by Johnson & Johnson, New Brunswick, USA) [8,9,10,11,12].

Similar to other microsomal P450 proteins, CYP17A1 also requires electrons supplied from reduced nicotinamide adenine dinucleotide phosphate (NADPH) through cytochrome P450 oxidoreductase (POR) (Figure 1) [2,13,14,15,16]. The 17,20 lyase activity of CYP17A1 is influenced by the presence of cytochrome b_5_ (CYB5A) in specific locations in different cells and tissues and guides the steroid hormone pathway in different directions [4] (Figure 2).

Along with CYB5A, higher molecular ratios of POR and phosphorylation of CYP17A1 also influence 17,20 lyase activity [17,18,19,20,21]. Recently several X-ray crystal structures of solubilized human CYP17A1 have been reported, but the structural basis of 17α-hydroxylase and 17,20, lyase activities remains unknown [22,23,24,25,26]. Generally, the mutations that affect the steroid-binding domain of CYP17A1 or disturb the interaction with P450 oxidoreductase (POR) for electron transfer, cause combined 17α-hydroxylase and 17,20 lyase deficiency, and are those more frequently found in humans [4,25]. Very few point mutations in CYP17A1 (R347C/H, R358Q) have been reported to cause isolated 17,20 lyase deficiency [27,28,29,30] (Table 1). These mutations are thought to interfere with CYB5A binding and/or electron transfer from POR to CYP17A1 during the 17,20 lyase reaction.

Lack of 17α-hydroxylase activity of CYP17A1 results in a compensatory overproduction of corticosterone and deoxycorticosterone with a weak glucocorticoid and significant mineralocorticoid action, which results in severe hypertension and hypokalemia. On the other hand, the 17,20 lyase deficiency results in a lack of sex steroids, leading to the 46, XY disorder of sexual development (DSD) with severe undervirilization in the “male” newborn, and deficient pubertal development and fertility in both sexes [4]. Previously, we have reported that disturbing the interaction of CYP17A1 with P450 oxidoreductase (POR) for electron transfer causes combined 17α-hydroxylase and 17,20 lyase deficiency [2,14,16,31,32,33,34]. Mutations identified on the surface of *CYP17A1* (R347C/H, R358Q) have been proposed to diminish the interaction with POR but could not explain the mechanism of their specific effect on 17,20 lyase activity [27,35].

Recently we have shown that in the earliest reported cases of apparent isolated 17,20 lyase deficiency, that were based solely on hormonal and morphological findings and without genetic analysis, the *CYP17A1* and *POR* genes were actually normal and mutations in *AKR1C2* and *AKR1C4* were found to cause a similar phenotype [36,37,38]. In the current report, we are describing a novel active site mutation in CYP17A1 that specifically abolishes the 17,20 lyase activity.

## 2. Results

### 2.1. Case Report and Genetic Analysis of the Patient

**Figure 3:**
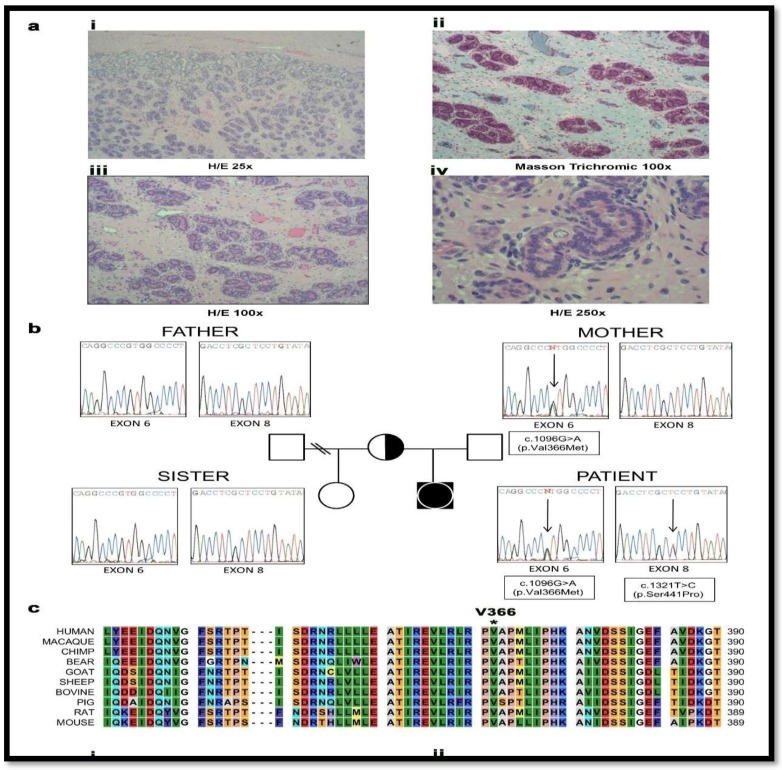
Genetic characterization and testis histology of the 46, XY disorder of sexual development (DSD) patient with a female phenotype. (**a**) Histopathologic studies revealed that both gonads consisted of atrophic and immature seminiferous tubules which were filled almost exclusively with pre-Sertoli cells. The fertility index was 5% with abortive and fetal type spermatogonia (i–iv); iii. in some areas, the tubules formed lobule-like structures similar to pseudo-hamartomas surrounded by collagen fibrous tissue; iv. nests of fibroblastic pre-Leydig cells were found in the interstitium. Albuginea and epidydimus were unremarkable. H/E-hematoxylin-eosin stain. (**b**) Family tree and electropherograms showing the index patient, the parents and the half-sister. Both, the patient and mother harbored the heterozygote c.1096G > A (p.Val366Met) mutation in exon 6, while the heterozygote c.1321T > C (p.Ser441Pro) mutation in exon 8 was only present in the patient. The father and the half-sister were both non-carriers of either mutation. (**c**) Alignment of human CYP17A1 amino acid sequence with a range of CYP17A1 proteins from other species found in the Universal Protein Knowledgebase (UniProt) database. The Valine 366 residue is highly conserved in all species studied**.**

**Figure 4:**
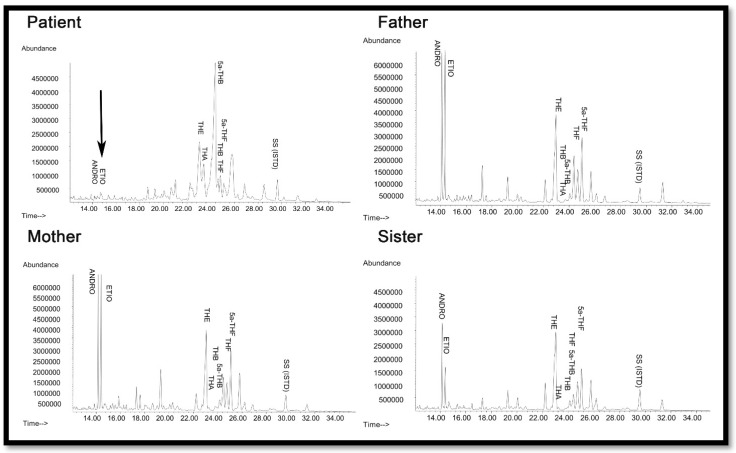
Urine steroid profile of the patient, heterozygote mother, father, and sister. The steroid analysis was performed on 24 h urine samples by GC/MS. Note the lack of androgens (ETIO, ANDRO) and the relative increase in corticosterone metabolites (THA, THB, 5α-THB) in the steroid profile of the patient compared to mother and father. THA—tetrahydro-11-dehydrocorticosterone; THB—tetrahydro corticosterone; 5a-THB-5α-tetrahydro corticosterone; THE—tetrahydrocortisone; THF—tetrahydrocortisol; 5a-THF-5α-tetrahydrocortisol; ETIO—etiocholanolone; ANDRO—androsterone; SS (ISTD)—internal standard.

The patient was born at term, with normal female external genitalia, after a normal spontaneous pregnancy, whereas an older sister was the product of an insemination with donor semen to avoid retinitis pigmentosa carried by the father’s family. At 2 months of age, the patient was operated for a right inguinal hernia. No female internal sex organs were found and karyotype was 46, XY. During the procedure, a gonad was detected and biopsied showing to be a testis (Figure 3). Electrolytes were normal and baseline hormone values at 3 months of age revealed moderately elevated ACTH, highly elevated PROG, normal/low 17OH-PREG, 17OH-PROG, 11-deoxycortisol and cortisol, undetectable androstenedione (Δ_4_A) and normal DHEA-S and Testosterone for female sex (Table 2). At the age of 5 months a human chorionic gonadotropin (hCG) test (500 IU/d × 3) was performed which showed no increase of Δ_4_A and T upon stimulation (Table 2). At 20 months of age an ACTH test (Synacthen^®^) revealed a moderately elevated baseline ACTH and a normal baseline plasma renin activity (PRA), Prog was highly elevated and further increased upon stimulation, whereas baseline 17OH-PREG, 17OH-PROG, cortisol, 11-deoxycortisol, and aldosterone were normal/low, and did not increase after stimulation (Table 2). Baseline DHEA-S and baseline and stimulated Δ_4_A were undetectable. Because of female phenotype and obvious biochemical lack of androgens, female sex of rearing was confirmed and a gonadectomy was performed at the age of 20 months. Testes morphology showed abnormal findings similar to those found in androgen-insensitive patients (Figure 4a). Blood pressure (BP) control was recommended as a precautionary measure while hydrocortisone replacement therapy was postponed depending on follow-up. BP controls revealed normal values. Baseline BP controls revealed normal values up to 11 years of age as well a 24-h ambulatory BP monitoring performed at 8 years of age (baseline and at the end of 3 months of therapy with hydrocortisone: 6 mg/m^2^ in 3 daily doses).

Initial genetic analysis of the genes for *AR* and *SRD5A2* were normal [39,40] and hormonal findings suggested partial 17α-hydroxylase and complete 17,20 lyase deficiencies (Table 2). The *CYP17A1* gene was analyzed and compound heterozygous point mutations c.1096G > A (V366M) and c.1321T > C (S441P) in exons 6 and 8 were identified (Figure 4b). The healthy fertile mother was found to carry the V366M mutation, a residue which is highly conserved across species (Figure 4c), while the father was normal. Therefore, *CYP17A1* S441P might be a de novo mutation in the patient although paternity testing was not performed. The mutations in the patient are likely to be on different alleles as even total disruption of one copy of *CYP17A1* does not result in disease. As both *CYP17A1* mutations have not been described previously, and as our patient presented with a very rare phenotype of apparent isolated 17,20 lyase deficiency, we performed further investigations to characterize these novel mutations.

### 2.2. Steroid Analysis

**Figure 5:**
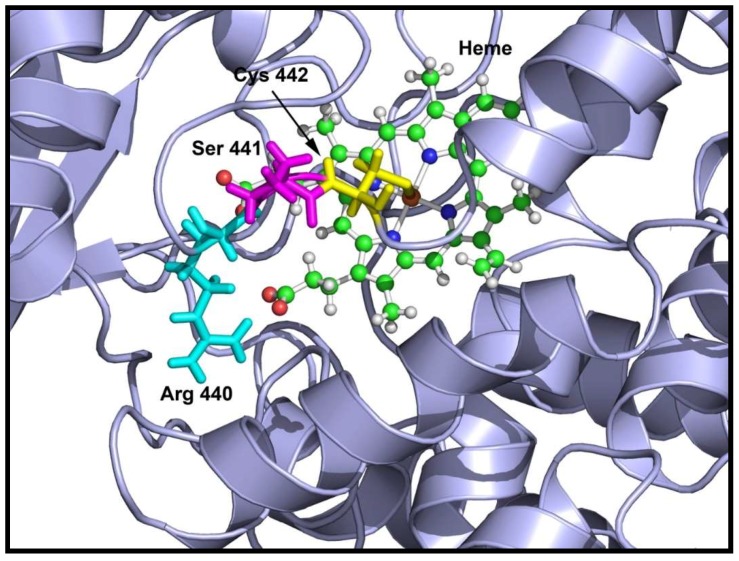
Location of S441P mutation in CYP17A1 structure showing the affected amino acids. The cysteine 442 is shown in yellow, serine 441 is in magenta and arginine 440 is in cyan. Both the cysteine 442 and arginine 440 are required for heme binding and the serine 441 to proline mutation creates a bend in the loop containing the cysteine 442 and arginine 440 residues, resulting in loss of heme binding that leads to an inactive enzyme.

Urine steroid profiling of the patient revealed almost complete loss of androgen metabolites, low-normal cortisol and elevated corticosterone metabolites (Figure 5), suggesting partial 17α-hydroxylase and complete 17,20 lyase deficiency. All other family members had normal steroid profiles (Figure 4).

### 2.3. Loss of 17,20 Lyase Activity of *CYP17A1* by the V366M Mutation

To investigate the molecular basis of these mutations we produced both mutant and the wild-type CYP17A1 proteins and performed enzyme kinetic assays (Table 3).

Comparison of mutant and wild-type proteins revealed that both mutations V366M and S441P affected CYP17A1 enzyme activities and therefore qualified as disease-causing mutations. The S441P mutation was found to cause a complete loss of both 17α-hydroxylase and 17,20 lyase activities of CYP17A1. Structural analysis of the S441P mutation indicated that heme binding may be affected (Figure 5), and quantification of heme in the S441P mutant revealed that it contained less than 5% of heme compared to the wild-type enzyme (Table 4).

The CYP17A1-V366M protein retained >40% of WT activity in the 17α-hydroxylase assay but had no activity in the 17,20 lyase assay using 17OH-PREG as substrate (Table 3). Therefore, S441P is a loss-of-function *CYP17A1* mutation whereas V366M qualifies as a rare mutation predominantly affecting CYP17A1-17,20 lyase activity *in vitro*. Therefore, the S441P mutation effectively created a non-functional allele and allowed us to explore the specific effects of the V366M mutation in greater detail.

### 2.4. The 17OH-PREG is Not an Effective Inhibitor of 17α-Hydroxylase Reaction by the V366M Mutant

For the human CYP17A1, PREG, 17OH-PREG, and PROG are all very good substrates and, therefore, are expected to compete for the binding to CYP17A1 when more than one substrate is present at the same time. To test if there is a difference between the WT versus the V366M mutant of CYP17A1, we used 17OH-PREG as an inhibitor for the 17α-hydroxylase reaction of CYP17A1 using radiolabeled [^3^H]PROG as substrate. For the WT CYP17A1, 17OH-PREG inhibited the 17α-hydroxylation of PROG with an observed IC_50_ value of 1.7 µM (Table 5).

For the V366M mutant of *CYP17A1*, no inhibition of the 17α-hydroxylation of PROG was observed by 17OH-PREG within the range of concentration used in the assay (0–100 µM). These results indicated difficulty in the binding of 17OH-PREG to the V366M mutant of CYP17A1. By contrast, when PREG was used as an inhibitor in the 17α-hydroxylation reaction catalyzed by CYP17A1 using PROG as a substrate, both the WT as well as V366M mutant enzymes were inhibited with apparent IC_50_ values of 0.9 µM for the WT enzyme versus 1.4 µM for the V366M mutant (Table 5). This suggests that both PREG and PROG can bind and be metabolized by the V366M variant of CYP17A1 but 17OH-PREG could not be used as a substrate by the mutant enzyme.

### 2.5. Computational Structural Analysis by Molecular Dynamics

**Figure 6:**
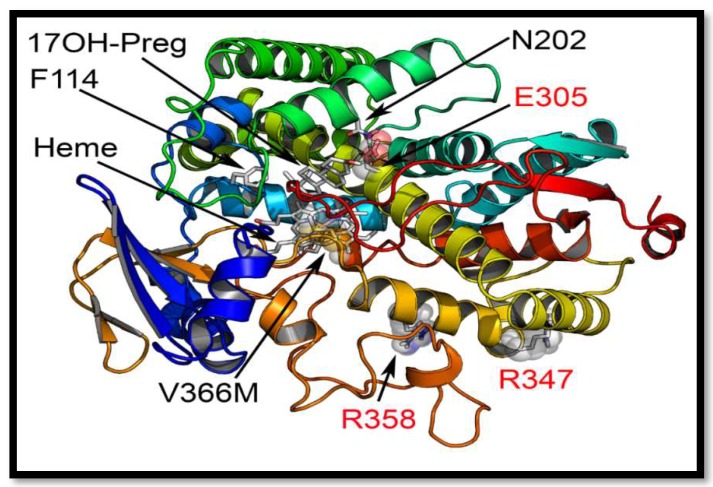
Comparison of novel V366M mutation with the previously reported isolated CYP17A1-17,20 lyase mutations. Only three other residues in CYP17A1 have been reported to be mutated in patients with isolated 17,20 lyase deficiency. The R347 and R358 are at the redox partner binding sites and their mutations may interfere with binding of POR and /or cytochrome b_5_. The E305 residue at the active site is important for orientation of the substrate and its mutation has been shown to alter substrate specificity and lead to a preference for progesterone as the more efficient substrate. The V366 is located exactly at the active site of the CYP17A1.

**Figure 7:**
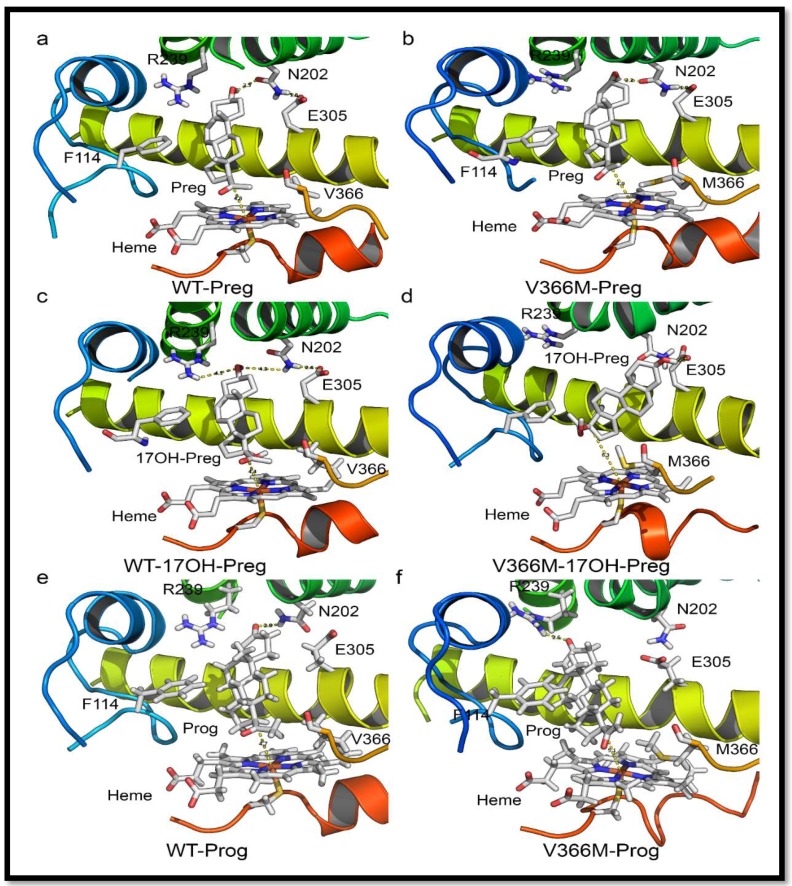
Structure analysis of the V366M mutation. (**a**) Binding of pregnenolone in CYP17A1 active site. Pregnenolone binds perpendicular to heme and is converted to 17OH-PREG. (**b**) Binding of pregnenolone to V366M variant of CYP17A1. Pregnenolone can still bind to V366M variant and is metabolized to 17OH-PREG. The side chain of methionine 366 in the mutant structure protrudes into the active site but still allows 17OH-PREG to exit. This is likely to slow down the reaction velocity of the 17α-hydroxylase reaction, which was confirmed by enzyme kinetic experiments (Table 1). (**c**) Binding of 17OH-PREG to WT CYP17A1. (**d**) Docking of 17OH-PREG to V366M variant of CYP17A1. The 17OH-PREG could not bind in the proximity to heme and distances between the heme iron and C17 atom increased for the mutant enzyme (6.3 Å in the mutant vs. 4.4 Å for the WT enzyme). Increased iron-C17 distances are observed together with N202-O3 interactions which are considered undesirable for optimal binding of 17hydroxy steroids for 17,20 lyase reaction. The methionine 366 side chain in the mutant protein blocks the entry of 17α-hydroxy steroid into the active site, resulting in loss of 17,20 lyase activity. (**e**,**f**) Comparison of WT and V366M variant of CYP17A1 binding to PROG.

We used the recently solved crystal structures of the human CYP17A1 [22,23] to make in-silico mutations and analyzed the changes through molecular dynamics (MD) simulations. In the CYP17A1 crystal structure, the active site for the binding of steroids is characterized by the positioning of residues V366, N202 and E305 (Figure 6). This led us to hypothesize that the specific space requirements exist for correct positioning of 17α-hydroxysteroids. The larger side chain of methionine in the V366M mutant protruded into the active site of CYP17A1 (Figure 7). Interestingly the shape of the protruding side chain of methionine 366 indicated that it might restrict the movement of steroids only in one direction, allowing the 17-hydroxy-steroids to leave the active site by the flexibility of movement in one direction, but creating a strong steric hindrance in the path of incoming steroids.

One of the most intriguing questions about CYP17A1 activities has been whether the 17α-hydroxysteroid can stay in the catalytic site and be metabolized again to androgen precursors, or whether the product of 17α-hydroxylase reaction leaves the active site and re-enters for the second reaction (Figure 7a). The one-way valve created by V366M mutation provides some answers to this question for the human CYP17A1. The presence of 17α-hydroxylase activity in the V366M variant suggested that PREG can get into the active site and be converted to 17OH-PREG (Figure 7b). If the 17OH-PREG was not able to leave the active site, it would have created an irreversible inhibition and the enzyme would have been unable to carry out further reactions. However, the 17α-hydroxylase reaction progressed (Figure 4 and Table 3), indicating that 17OH-PREG can exit the active site. Furthermore, if the 17OH-PREG could be converted without exiting the active site, then DHEA formation should have been observed as in case of the wild-type enzyme.

However, DHEA was absent in both the *in vitro* enzyme reactions of the V366M mutant of CYP17A1 (Table 3) as well as in the urine of the patient (Figure 4), clearly indicating that 17OH-PREG must exit the active site after the 17α-hydroxylase reaction. If the problem was with the exit of DHEA, then that would also have created an irreversible inhibition by blocking the active site with one molecule of DHEA per unit of enzyme and further enzymatic activity would have come to a halt, but that was clearly not the case from the *in vitro* enzyme analysis as well as the patient’s urine steroid profile (Figure 4 and Table 3). In the case of bovine CYP17A1, about 20% of pregnenolone consumed in the reaction could be converted to DHEA without exiting the active site [41]. When PROG was used as a substrate, compared to 17OH-PREG the dissociation of 17OH-PROG was 10 times faster. The release of the intermediate reaction was much faster than 17,20 lyase reaction and that prevented the direct formation of androstenedione from PROG. There are significant differences in activities of CYP17A1 between different species and the human enzyme has a very poor affinity for 17OH-PROG as substrate [25,42,43].

**Figure 8:**
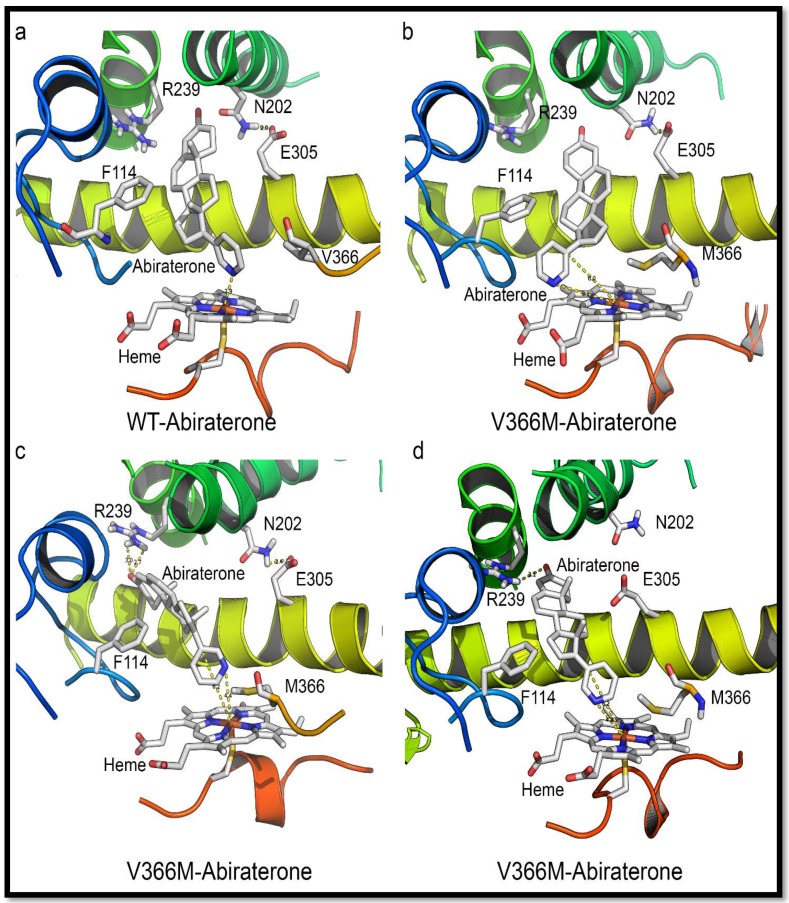
Comparison of abiraterone interaction with the WT and V366M variant of CYP17A1. (**a**) In the WT CYP17A1 abiraterone binds by forming a nitrogen-iron co-ordination (2.9 Å) with the central heme. (**b**–**d**) Different poses of abiraterone seen during docking into the V366M mutant of CYP17A1. In the V366M mutant, the methionine side chain prevents the binding of abiraterone and increased distances between the C17 atom and heme iron are observed together with binding poses that are different from the WT.

To further study this mechanism, we employed molecular dynamics simulations for the analysis of steroid binding in the active site of wild-type and the V366M mutant of CYP17A1. This was confirmed by the ensemble docking experiments which (for wild-type protein) yielded ligand poses in close proximity to the heme and highly resembling the co-crystallized abiraterone in the *CYP17A1* (PDB: 3RUK) structure [22]. The validity of our docking protocol was supported by a set of additional CYP17A1 crystal structures containing all the ligands of interest (PDB codes: 4NKW, 4NKX, 4NKY, and 4NKZ) in very similar poses enabling hydrogen-bonding to a distal N202 residue for PROG/PREG but not for 17OH-PREG [23]. Based on these observations, we hypothesized that the methionine side-chain could prevent a closer proximity to the heme iron necessary for the substrates undergoing lyase reaction [23,24]. To check this, we performed an additional pair of simulations with 17OH-PREG docked into the wild-type and mutant binding sites. Thus, we could indeed measure that the average distance between the ligand C17 atom and the heme iron differed in these systems (4.4 Å for wild-type and 6.3 Å for V366M mutant) (Figure 8c,d). While these results cannot be compared directly to the crystal structure of CYP17A1 with 17OH-PREG (PDB code 4NKZ; the O17-Fe distance between 3.4 and 3.9 Å depending on the chain), this hypothesis is well in line with all our experimental data and recently reported structures of human CYP17A1 in complexes with steroid substrates. In addition, the simulations, as well as the docking to the mutant protein, provided a possible explanation for the decreased reaction rates of the pregnenolone hydroxylation. Specifically, these structures (Figure 7b,d,f) hinted at an electrostatic interaction between the methionine methyl group (positively polarized by the preceding sulfur atom) and the negatively-charged carboxylate found in all ligands we studied. We propose that this added interaction slows down ligand dissociation/exit and thus slows down the reaction. Our MD simulation and docking results agree with greatly reduced Vmax but only marginally increased Km of both the wild-type and V366M mutant of CYP17A1 for the PROG (Table 3), indicating that binding of PREG and PROG is not affected but the exit of the 17α-hydroxy steroid from the active site may be slower in the V366M variant.

### 2.6. Substrate- and Inhibitor-Binding Analysis

Abiraterone bound to the WT CYP17A1 with a Kd value of 95 nM and showed the typical soret peak at 427 nm, indicative of nitrogen co-ordination with heme iron (Table 5). However, in case of the V366M mutant of CYP17A1, this binding pattern was not observed. To test the integrity of the protein, we used imidazole to check the binding and found that imidazole itself could bind to the V366M mutant. This confirmed our hypothesis that the introduction of the bulky methionine to replace the valine at position 366 creates steric hindrance and perturbs the binding pattern of drugs and steroids. These results also point to structural considerations for future inhibitor development which may utilize the spatial arrangement of binding-site residues to design tight-fitting inhibitors. Progesterone bound with slightly decreased affinity to the V366M mutant (Kd 287 nM compared to 162 nM for the WT enzyme). Binding of 17OH-PREG was not observed for the V366M mutation and explained the lack of 17,20 lyase activity, while for the WT CYP17A1 a Kd value 142 nM was observed. It is possible that some apparent binding of 17OH-PREG may occur at exceedingly high, non-physiological levels due to the nature of the structural hindrance from the V366M mutation which is at the very end of the binding site and close to the heme iron and water molecules that occupy the binding pocket.

Since the spectral binding of steroid substrates to CYP17A1 is observed by the replacement of water molecules at the active site, it is possible for some apparent binding to emerge from titration studies using very high enzyme and substrate concentrations, but due to unfavorable binding poses and distances, as revealed by further computational structure analysis, such apparent binding is not likely to result in an enzyme-substrate complex that can lead to product (DHEA) formation. This is further evidenced by lack of inhibition by 17OH-PREG in the 17hydroxylation reaction using PROG as substrate. In the case of inhibitor abiraterone, which needs to form an iron-nitrogen co-ordination and gets much closer to heme iron than steroid substrates (2.9 Å compared to 4.4–4.8 Å) (Figure 8 and Figure S1), no binding was observed. Taken together these data indicate a steric hindrance for the binding of 17OH-PREG which may result in poor interaction with the enzyme and loss of 17,20 lyase activity.

### 2.7. Mechanism of Steroid/Abiraterone Binding and Action in Relation to the V366M Mutation

**Figure 9:**
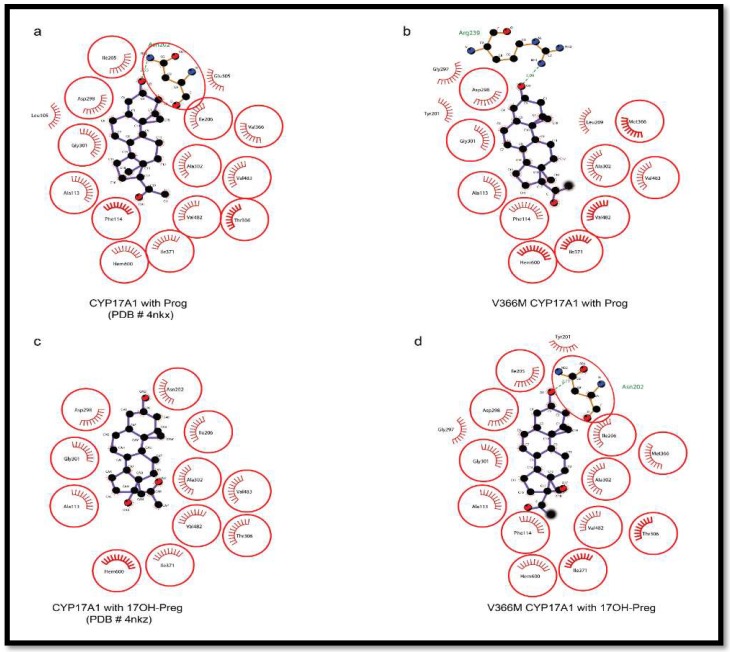
Comparison of PROG and 17OH-PREG bound in crystal structures of human CYP17A1 with the docked steroids into the V366M mutant. (**a**,**b**) PROG is found to bind similarly to WT as well as the mutant enzyme. (**c**,**d**) Mutation of V366 to M leads to non-optimal interactions with the N202 group in the V366M mutant compared to WT where this interaction is absent in poses which have 17OH-PREG close to the heme.

Docking was performed with all ligands to WT as well as the V366M mutant of CYP17A1. Thr306 is reported to be a vital residue for the hydroxylation reaction (part of an alcohol pair donating protons). It is also very close to the heme and, like residue 366, is near the bound ligands. Asn202 is reported to interact with the distal side of the molecules. It is at the end of the cavity formed around the bound steroids/abiraterone (Figure 6) and most likely one of the residues inside the access channel. Ensemble docking of all relevant ligands implies that docking poses mimic the co-crystallized abiraterone (CH3 groups to the left when looking from the direction of residue 366) and allow interaction with the residue 202 highlighted in recent structures of human CYP17A1 (Figure 8 and Figure 9) [23].

The pose from which 17α-hydroxy steroid leaves the site (after being formed) may differ from the one it assumes upon coming back. There are also possible differences between 17OH-PREG and PROG/PREG binding (Figure 7 and Figure 9). Angular analysis of apo and relaxation V366M simulations imply that the methionine moves around a single conformation (chi2 and 3 angles roughly in 20-degree windows—a good match to the rotamers reported by http://www.dynameomics.org). It seems most likely that the CH3 group is involved in steric effects. The sulfur group in methionine may further polarize it, enhancing interaction with the carboxylate of 17α-hydroxy steroids. In some MD poses the methionine could be seen moving away from the heme, indicating it has conformational flexibility and in the presence of the metabolized PREG/PROG the 17α-hydroxylated ligand may exit without excessive hindrance. The reduced velocity of the PROG to 17OH-PROG reaction indicates that there could be an interaction between the carboxylate and the (likely-polarized) CH3 group of the methionine, which may slow down the exit of the product. Some other differences in the binding pattern were observed, like the involvement of Arg239 in the interaction of PROG with the V366M variant which may create bottlenecks during the exit of the product. Computationally calculated binding constants and binding energy calculations also supported the experimental results of reduced binding affinities for 17OH-PREG and abiraterone for the V366M mutant (Table 6). All this implies a general steric hindrance by the bigger residue (methionine vs. valine).

Given the information from crystal structure of human CYP17A1 with abiraterone (nitrogen at 2.9 Å from the heme iron) (Figure 8), this implies that PREG either does not have to get this close to the heme or can accesses it from a different angle and is hence relatively unimpaired by the methionine. Recent structures of the human CYP17A1 bound with different steroid substrates confirm this hypothesis and show that 17OH-PREG binds much closer to the heme iron than PROG or PREG (Figure 7 and Figure 9) [23,24]. For the 17OH-PREG we observed the existence of poses with the hydroxyl positioned very close to the CH3 of the methionine, indicating the bulky methionine residue could be preventing the ligand from getting close enough to the heme (Figure 7d and Figure S2).

In addition, we also observed shorter distances suitable for hydrogen bonds with N202 and R239 groups and 17OH-PREG in the docking poses of V366M mutant (2.6 Å compared to 4.4 Å for the WT) (Figure 7d and Figure S2, Table 6); it has been proposed that these interactions are required for PROG/PREG but are considered suboptimal for 17,20 lyase reaction and are found together with increased distances of C17 in 17α-hydroxy steroid substrate and the heme iron. Increased distances between the C17 of 17OH-PREG/Abiraterone and the heme iron, together with the extra/undesired interactions with N202/R239, were consistently observed in our simulations (Figure 7c, Figure 8, Figures S1 and S2).

It is more likely that the ligands cannot get into the binding site rather than have too much difficulty in leaving it since that would block the active site and prevent the 17α-hydroxylation reaction from occurring (which is not severely affected as observed by enzymatic analysis as well as the urine steroid profile of the patient). Also, DHEA is the smallest of all steroids involved, making it the most likely to leave, so it is the entry of 17OH-PREG into the active site that is likely to be impaired. Many different poses of abiraterone and 17OH-PREG were observed for the V366M mutant which illustrates the non-optimal binding pattern for the mutant enzyme (Figure 8 and Figure S2). The inhibitor abiraterone (similar scaffold but bigger than the steroid substrates) also has trouble binding (Figure 8 and Figure S1, Table 5) and does not act as an inhibitor for the 17α-hydroxylation reaction catalyzed by the V366M mutant of *CYP17A1* (Table 5).

## 3. Discussion

Among the previously reported mutations causing isolated 17,20 lyase deficiency, the R347C, R347H and R358Q are located on the redox partner binding site (Figure 6) and seem to act by altering the interaction with POR. Generally, a defect in redox partner or loss of interaction with redox partner affects all cytochrome P450 activities in the endoplasmic reticulum as well as in mitochondria [31,36,44,45,46,47,48]. In the case of R347H and R358Q mutations in CYP17A1, why this loss of interaction with POR affects the 17,20 lyase activity in a more severe fashion than the 17α-hydroxylase activity is not clear, since both activities of CYP17A1 require electrons supplied by NADPH through POR for their catalytic function [49]. The only other mutation in CYP17A1 that had been reported to selectively impair the 17,20 lyase activity is E305G which is part of the substrate access channel of CYP17A1 (Figure 6 and Figure 8) [28,30]. The E305G mutation had been reported to affect the binding of 17α-hydroxy steroids while showing even higher than normal 17α-hydroxylase activities [28]; however a later analysis of the patient’s steroid metabolic profile [30] showed that 17α-hydroxylase activity was also impaired, contradicting the earlier claims of isolated 17,20 lyase deficiency [30]. A more efficient coupling with POR and efficient use of NADPH have recently been proposed to favor the 17,20 lyase reaction [49]. Multiple potential mechanisms for the selectivity of the 17,20 lyase reaction have been proposed in recent works from different laboratories [23,26,49,50,51,52,53].

The V366M is an active site mutation in CYP17A1 that not only preferentially targets 17,20 lyase activity but also provides insights into the structural basis of the 17,20 lyase reaction, the key regulator of sex steroid biosynthesis in humans (Figure 2 and Figure 7). The specificity of the human CYP17A1 active site allows the binding of PREG and its 17hydroxy metabolite (17OH-PREG) to bind in different conformations and exit the active site after the reactions (Figure 7a,c), and the mutation of valine 366 to methionine alters the active site and hinders the binding of 17α-hydroxy-steroids (Figure 8d). The V366M mutant also explains the effectiveness of the anti-prostate-cancer drug abiraterone as a potent inhibitor of CYP17A1. Abiraterone fills the active site of CYP17A1 (Figure 8 and Figure S1) and becomes irreversibly bound to the enzyme (Supplementary Figure S1c), stopping any further substrate binding and activity [22]. This location of V366 at the catalytic center provides a structural basis for improving and designing novel and specific CYP17A1 inhibitors by targeting spatial selectivity of the active site with imidazole or other suitable chemical moieties added to core steroid structures.

Structural modifications based on the above information may help in designing more specific and potent inhibitors directed only towards the 17,20 lyase activity of CYP17A1 that could produce tighter binding at the active site. The 17,20 lyase-specific inhibitors will have advantages over current compounds that target both the 17α-hydroxylase and the 17,20 lyase activities of CYP17A1 and require steroid supplementation [12,54,55,56,57,58]. The mechanistic and structural insights revealed by these studies will help in the development of better drugs against polycystic ovary syndrome and prostate cancer. These findings will also improve our understanding of the structural basis of the dual function of CYP17A1 as both a 17α-hydroxylase and 17,20 lyase, and the role of these activities in the regulation of steroid hormone production in different tissues.

## 4. Materials and Methods

### 4.1. Human Subjects

All clinical investigations were carried out following the Declaration of Helsinki principles. Written informed consent was received from the parents for genetic work-up of 46, XY DSD in their child in the pediatric endocrinology research laboratory in Barcelona which holds ethical approval for these studies.

### 4.2. Genetic Analysis

DNA was extracted from the peripheral blood leucocytes of the patient. Genetic analysis of the androgen receptor gene (*AR*) and for the 5αreductase type 2 gene (*SRD5A2*) were performed as described [39,40], yielding normal results. The *CYP17A1* gene was analyzed as reported [59] and identified sequence variations were compared to National Center for Biotechnology Information (NCBI, Bethesda, USA) entry NG_007955.1 (GI:189339218). The *CYP17A1* gene was subsequently also analyzed in the parents and the older half-sister, and one mutation was found in the mother. Since the second mutation is a completely inactivating mutation, its presence on a different allele was inferred (as the presence of both mutations on one allele will give a good working copy of the *CYP17A1* gene, and that does not cause disease).

### 4.3. Steroid Profiling from 24-h Urine Samples

Steroid metabolites in urine were measured by the gas chromatography-mass spectrometry (GC/MS) method as described previously [60,61].

### 4.4. Recombinant Protein Expression

Human wild-type and mutant *CYP17A1* proteins were produced in an *E. coli* expression system and purified for enzyme kinetic assays [15,17,18,62]. The pCWH17-mod(His)4 expression plasmid (a gift from Prof. Michael Waterman, Nashville, TN) containing the cDNA for human WT or mutant CYP17A1 [15], was transformed into the *E. coli* JM109 cells and colonies were selected under ampicillin control. Bacteria were grown at 37 °C to OD_600_ 0.6 and the CYP17A1 protein expression was induced by the addition of 0.5 mM IPTG followed by further incubation at 28 °C for 48 h. Purification of CYP17A1 was performed as described [15,17,18]. In brief, the spheroplasts prepared by the lysozyme treatment of bacterial cells were ruptured by sonication and cleared by centrifugation at 4000× *g* for 10 min, then the supernatant containing the CYP17A1 protein was extracted with 1.5% Triton X-114 and centrifuged at 100,000× *g* for 30 min. A reddish-brown colored detergent-rich supernatant fraction containing the CYP17A1 was isolated, diluted to reduce the detergent concentration to 0.1%, and passed over a Ni-NTA-sepharose column. The column was washed with 5 mM histidine to remove the non-specific binding and eluted with 200 mM histidine. Further purification was carried out by gel filtration chromatography to remove histidine and other protein contaminants.

### 4.5. In Vitro Enzyme Kinetic Analysis of Identified CYP17A1 Mutations

To assess 17α-hydroxylase activity, 10 pmol of CYP17A1 along with 20 pmol purified human POR [17,31,32,34,62] (at a 1:2 ratio) was incubated with 0.1–15 μM [^14^C]PROG (80,000 cpm/reaction) and 1 mM NADPH in 50 mM potassium-phosphate buffer (pH 7.4) containing 6 mM potassium acetate, 10 mM MgCl_2_, 1 mM reduced glutathione, 20% glycerol and 20 µg phosphatidylcholine for 60 min at 37 °C. Human CYP17A1 does not use 17OH-PROG as a major substrate and, therefore, PROG is considered a better substrate to monitor only 17α-hydroxylase activity due to very little further conversion of 17OH-PROG. To assess 17,20 lyase activity, CYP17A1 and POR proteins were incubated with 0.05–5 μM [^3^H]17OH-PREG (100,000 cpm/reaction), 1 mM NADPH and 20 pmol cytochrome b_5_/reaction in 50 mM K-phosphate buffer (pH 7.4) containing 6 mM potassium acetate, 10 mM MgC_l2_, 1 mM reduced glutathione, 20% glycerol and 20 µg phosphatidylcholine for 90 min at 37 °C. Steroids were extracted and resolved by thin layer chromatography before quantitative analysis for conversion to 17OH-PREG and DHEA respectively as described [19]. For inhibition assays, abiraterone, PREG as well as 17OH-PREG were used to compete with radioactive PROG for 17α-hydroxylase reaction. Enzyme kinetic calculations were performed using non-linear regression curve fitting with Prism (GraphPad Software Inc., San Diego, CA, USA). Data represent the mean of three independent experiments.

### 4.6. Heme and P450 Measurements

Heme content was measured as described previously by dissolving the protein in NaOH and measuring the heme absorbance in a triton-methanol mixture [63]. Cytochrome P450 and Cytochrome b_5_ were measured as described previously [1,17]. Cytochrome b_5_ content was estimated by monitoring the absorbance difference at 423–490 nm using an extinction coefficient of 181 mmol cm^−1^.

### 4.7. Substrate-Binding Assay

Binding of steroid substrates to CYP17A1 was measured by recording the substrate-binding spectra in the range of 340–500 nm on a Perkin Elmer Lambda 25 spectrophotometer. A change in heme absorbance is observed when water molecules at the active site are replaced by steroid substrates [22]. In case of abiraterone, an increase in the soret peak at 427 nm is observed by co-ordination of the heme iron with pyridine nitrogen. To accurately measure the tight binding substrates and inhibitors, a cuvette with 100 mm path length was employed and protein concentration was kept at 50 nM. Binding of steroids, as well as abiraterone to CYP17A1, occurs with very high affinity and, as observed previously, Kd values measured are often close to protein concentration used in binding assays. All substrates and abiraterone were dissolved in ethanol and an equal amount of ethanol was added to the reference cuvette and the final concentration of ethanol added to the cuvettes was kept below 2%. After each addition of the compounds, cuvettes were incubated for 5 min at 22 °C before recording the spectra. The slit width was fixed at 1.0 nm and spectra were recorded at 50 nm/min with a series of increasing concentrations of different compounds (abiraterone: 0–200 nM; PREG: 0–500 nM; 17OH-PREG: 0–1000 nM; PROG: 0–1000 nM). Data were fitted with GraphPad Prism based on the tight binding pattern of substrates to a single binding site.

### 4.8. Protein Structure Analysis of WT and Mutant CYP17A1

The published 3D structures of human CYP17A1 [22,23] were downloaded from the protein structure repository (www.rcsb.org). We performed several rounds of multiple-sequence alignments with different CYP17A1 protein sequences from several organisms and created in-silico mutants using the programs YASARA [64] and WHATIF [65]. For all further experiments described, a 2.6 Å resolution crystal structure [22] (PDB code 3RUK) of CYP17A1 was used; with the abiraterone ligand, the membrane anchor (all residues preceding R45), and water molecules (aside from one conserved between residue 366 and the heme) removed. Missing hydrogen atoms were added to the structure with YASARA [64] which was also used for all other computations. The V366M mutant model was then constructed by replacing the original valine with the most favorable methionine conformation found with the in-built SCWALL method (rotamer library search followed by energy minimization) [66]. Afterward, both systems were subjected to 10 ns explicit solvent MD simulations at 310 K, which was preceded by 500 steps of steepest descent and simulated annealing minimization using the AMBER03 force field and the TIP3P water model [67,68]. All following MDs were performed with similar settings. The resulting simulation snapshots (100 per run) were used for the docking of steroids with the AutoDock Vina [69] ensemble-docking experiments using PREG, 17OH-PREG, PROG and 17OH-PROG (orthorhombic docking was grid-established around the central heme and the residues 105, 202, and 366 of the CYP17A1). The final selection of poses was based on their docking scores and similarities to the abiraterone ligand which was co-crystallized in the template structure (PDB: 3RUK) [22]. The docked steroid poses agreed with the binding site details highlighted by a recent set of CYP17A1 crystal structures [23]. Clustering of simulation snapshots was done using USCF Chimera [70]. Figures of the structure models were created with the program Pymol (www.pymol.org) and the chosen poses were rendered as ray-traced images with POVRAY (www.povray.org). Ligand interactions were analyzed and depicted with the program LIGPLOT+ (http://www.ebi.ac.uk/thornton-srv/software/LigPlus/).

## Figures and Tables

**Table 1:** Reported cases of *CYP17A1* mutations causing isolated 17,20 lyase deficiency [27,28,29,30]. The mutation E305G which was initially reported by Sherbet et al. [28] to cause isolated 17,20 lyase deficiency, was later reported by Tiosano et al. [30] to also result in combined 17α-hydroxylase/17,20 lyase deficiency, similar to other common mutations in *CYP17A1* [30].

17OH Steroids Basal	17OH Steroids Stimulated	Cortisol Basal	Cortisol Stimulated	Activities (% of WT)		Ref
				17OHase	17,20 lyase	
Normal	Hyperresponsive	Normal	Areactive	65	5	Geller 1997 [27]
Elevated	Hyperresponsive	Normal	Areactive	65	5	Geller 1997 [27]
Slightly elevated	Not reactive	Low normal	Areactive			van den Akker 2002 [29]
Normal	Normal	Low normal	Areactive			van den Akker 2002 [29]
Normal/Elevated	Reactive	Low normal	Hyporeactive	60	0	van den Akker 2002 [29]
Normal/Elevated	Reactive	Low normal	Hyporeactive	60	0	van den Akker 2002 [29]
Normal/Elevated	Reactive	Low	Hyporeactive	100	0	Sherbet 2003 [28]
Normal/Elevated	Normal	Low normal	Hyporeactive			Tiosano 2008 [30]
Normal/Elevated	Normal	Low normal	Hyporeactive			Tiosano 2008 [30]
Normal/Elevated	Normal	Low normal	Hyporeactive			Tiosano 2008 [30]
Normal/Elevated	Normal	Low normal	Hyporeactive			Tiosano 2008 [30]
Normal/Elevated	Normal	Low normal	Hyporeactive			Tiosano 2008 [30]
Normal/Elevated	Normal	Low normal	Hyporeactive			Tiosano 2008 [30]
Normal/low	Hyporeactive	Low normal	Areactive	43	0/0	This report

**Table 2:** Laboratory findings in our patient harboring compound heterozygote *CYP17A1* mutations.

Age		3 Months		5 Months		20 Months			5 Years	6.5 Years	
Parameter	Unit	Basal	*Normal Range*	hCG Test (500 IU/d × 3)	*Normal Range*	Basal	*Normal Range*	ACTH-Stimulated	Basal	Basal	*Normal Range*
Sodium	mEq/L	140	136–145	-		141	136–145		-	144	136–145
Potassium	mEq/L	4.0	3.5–5.1	-		4.6	3.5–5.1		-	4.7	3.5–5.1
ACTH	pg/mL	81	9–50	82	9–50	66	9–50	-	62	46	9–50
Progesterone	ng/dL	222	5–80	-		251	10–50	425	522	339	10–50
17OH Preg	ng/dL	132	60–830	27	60–830	13	10–50	-	-	-	
17OHProg	ng/dL	300	40–460	40	40–460	29	19–159	35	110	80	10–470
DHEA-S	µg/dL	<5	5–62	<5	5–62	<5	5–190	-	<5	<5	5–95
11-Deoxycortisol	ng/dL	1100	1450 ± 790	200	1450 ± 790	140	186 ± 116	-	87	-	205 ± 108
Cortisol	µg/dL	3.7	4.3–22.2	8.6	4.3–22.2	8.9	4.3–22.2	7.8	7.1	4.1	4.3–22.4
Δ4A	ng/dL	<30	63 ± 39	<3	63 ± 39	<30	30–330	<30	-	<30	30–330
Testosterone	ng/dL	7	140 ± 132	<4	140 ± 132	28	15–30	16	-	-	
LH	IU/L	-		-		0.3	0.2–1.0	-	<0.07	-	0.2–1.0
FSH	IU/L	-		-		9	0.4–2.0	-	4.3	-	0.4–2.0
PRA	ng/mL/h	-		-		1.8	0.6–21.3	-	-	0.1	0.3–6.4
Aldosterone	ng/dL	-		-		12.1	14–114	11.5		6.2	9–66

**Table 3:** Kinetic parameters for the metabolism of PROG to 17OH-PROG (17hydroxylation) and 17OH-PREG to DHEA (17,20 lyase reaction) by WT and V366M mutant of CYP17A1.

CYP17A1 Variant	17α-HydroxylasePROG to 17OH-PROG			17,20 Lyase17OH-PREG to DHEA	
	Km (µM)	Vmax (min^−1^)	Cat Eff (%)	Km (µM)	Vmax (min^−1^)
**WT**	6.1 ± 0.7	0.71	100	0.92 ± 0.07	0.025 ± 0.004
**V366M**	8.4 ± 0.9	0.42	43	-	-
**S441P**	-	-	-	-	-

**Table 4:** Heme content of proteins used in assays.

Protein Preparation	Heme Content (nnol/nmol of Protein)
CYP17A1 WT	0.93
CYP17A1_V366M	0.95
CYP17A1-S441P	<0.05
CYB5A	1.0

**Table 5:** Binding and inhibition studies of WT and the V366M mutant of *CYP17A1*.

	CYP17A1_WT	CYP17A1_V366M
Binding studies	Kd (nM)	Kd (nM)
Binding of PROG	163 ± 29	287 ± 35
Binding of PREG	62 ± 17	92 ± 15
Binding of 17OH-PREG	142 ± 38	-
Binding of Abiraterone	85 ± 23	-
**Inhibition studies**	**IC_50_ (µM)**	**IC_50_ (µM)**
Inhibition of PROG 17α-hydroxylation by Abiraterone	0.04 ± 0.01	-
Inhibition of PROG 17α-hydroxylation by 17OH-PREG	1.7 ± 0.2	-
Inhibition of PROG 17α-hydroxylation by PREG	0.9 ± 0.15	1.4 ± 0.2

**Table 6:** Computational binding energy, computationally calculated dissociation constants, and interacting residues for steroid substrates and abiraterone with WT and the V366M mutant of *CYP17A1*. Key residues identified in different studies are shown in bold.

*CYP17A1* Protein	Binding Energy (kcal/mol)	Dissociation Constant (nM)	Contacting Residues
WT with Prog	10.6	14.66	ALA113 PHE114 **ASN202** ILE205 ILE206 LEU209 **ARG239** GLY297 ASP298 GLY301 ALA302 THR306 ALA367 ILE371 VAL482 VAL483 **HEME**
M366 with Prog	9.75	43.71	ALA113 PHE114 **ASN202** ILE205 ILE206 LEU209 **ARG239** GLY297 ASP298 GLY301 ALA302 THR306 **MET366** ALA367 ILE371 VAL482 VAL483 **HEME**
WT with Preg	10.7	13.29	ALA105 ALA113 PHE114 ILE205 ILE206 LEU209 VAL236 **ARG239** GLY297 ASP298 GLY301 ALA302 **GLU305** THR306 **VAL366** ILE371 VAL482 VAL483 **HEME**
M366 with Preg	10.3	27.11	ALA105 SER106 ALA113 PHE114 ILE205 ILE206 LEU209 VAL236 **ARG239** GLY297 ASP298 GLY301 ALA302 THR306 **MET366** ILE371 VAL482 VAL483 **HEME**
WT with 17OH-Preg	11.3	5.22	ALA113 PHE114 TYR201 **ASN202** ILE205 ILE206 LEU209 LEU214 **ARG239** GLY297 ASP298 GLY301 ALA302 THR306 **VAL366** ALA367 ILE371 VAL482 VAL483 **HEME**
M366 with 17OH-Preg	7.0	60.4	ALA113 PHE114 TYR201 **ASN202** ILE205 ILE206 LEU209 **ARG239** GLY297 ASP298 GLY301 ALA302 **GLU305** THR306 **MET366** ALA367 ILE371 VAL482 VAL483
WT with Abiraterone	12.5	0.69	ALA113 PHE114 TYR201 **ASN202** ILE205 ILE206 LEU209 **ARG239** GLY297 ASP298 GLY301 ALA302 **GLU305** THR306 **VAL366** ALA367 LEU370 ILE371 VAL482 VAL483 **HEME**
V366 with Abiraterone	8.1	1112.9	ALA105 SER106 ASN107 ALA113 PHE114 TYR201 ILE205 ILE206 LEU209 **ARG239** THR294 GLY297 ASP298 GLY301 ALA302 THR306 **MET366** ILE371 VAL482 VAL483

## Supplementary Materials

The following are available online at http://www.mdpi.com/1424-8247/11/2/37/s1, Supplementary Figures S1 and S2.

## References

[1] Omura T., Sato R. (1964). The carbon monoxide-binding pigment of liver microsomes. I. Evidence for its hemoprotein nature. J. Biol. Chem..

[2] Pandey A.V., Flück C.E. (2013). NADPH P450 oxidoreductase: Structure, function, and pathology of diseases. Pharmacol. Ther..

[3] Zanger U.M., Schwab M. (2013). Cytochrome P450 enzymes in drug metabolism: Regulation of gene expression, enzyme activities, and impact of genetic variation. Pharmacol. Ther..

[4] Miller W.L., Auchus R.J. (2011). The molecular biology, biochemistry, and physiology of human steroidogenesis and its disorders. Endocr. Rev..

[5] Zuber M.X., Simpson E.R., Waterman M.R. (1986). Expression of bovine 17 alpha-hydroxylase cytochrome P-450 cDNA in nonsteroidogenic (COS 1) cells. Science.

[6] Chung B.C., Picado-Leonard J., Haniu M., Bienkowski M., Hall P.F., Shively J.E., Miller W.L. (1987). Cytochrome P450c17 (steroid 17 alpha-hydroxylase/17,20 lyase): Cloning of human adrenal and testis cDNAs indicates the same gene is expressed in both tissues. Proc. Natl. Acad. Sci. USA.

[7] Nakajin S., Shinoda M., Haniu M., Shively J.E., Hall P.F. (1984). C21 steroid side chain cleavage enzyme from porcine adrenal microsomes. Purification and characterization of the 17 alpha-hydroxylase/C17,20-lyase cytochrome P-450. J. Biol. Chem..

[8] Vasaitis T.S., Bruno R.D., Njar V.C. (2011). CYP17 inhibitors for prostate cancer therapy. J. Steroid Biochem. Mol. Biol..

[9] Attard G., Reid A.H.M., Auchus R., Hughes B.A., Cassidy A.M., Thompson E., Oommen N.B., Folkerd E., Dowsett M., Arlt W. (2012). Clinical and biochemical consequences of CYP17A1 inhibition with abiraterone given with and without exogenous glucocorticoids in castrate men with advanced prostate cancer. J. Clin. Endocrinol. Metab..

[10] De Bono J.S., Logothetiset C.J., Molinaal A., Fizazi K., North S., Chu L., Chi K.N., Jones R.J., Goodman O.B., Saad F. (2011). Abiraterone and Increased Survival in Metastatic Prostate Cancer. N. Engl. J. Med..

[11] Attard G., Reid A.H., Yap T.A., Raynaud F., Dowsett M., Settatree S., Barrett M., Parker C., Martins V., Folkerd E. (2008). Phase I clinical trial of a selective inhibitor of CYP17, abiraterone acetate, confirms that castration-resistant prostate cancer commonly remains hormone driven. J. Clin. Oncol..

[12] Malikova J., Brixius-Anderko S., Udhane S.S., Parween S., Dick B., Bernhardt R., Pandey A.V. (2017). CYP17A1 inhibitor abiraterone, an anti-prostate cancer drug, also inhibits the 21-hydroxylase activity of CYP21A2. J. Steroid Biochem. Mol. Biol..

[13] Lu A.Y., Junk K.W., Coon M.J. (1969). Resolution of the cytochrome P-450-containing w-hydroxylation system of liver microsomes into three components. J. Biol. Chem..

[14] Flück C.E., Tajima T., Pandey A.V., Arlt W., Okuhara K., Verge C.F., Jabs E.W., Mendonça B.B., Fujieda K., Miller W.L. (2004). Mutant P450 oxidoreductase causes disordered steroidogenesis with and without Antley-Bixler syndrome. Nat. Genet..

[15] Imai T., Globerman H., Gertner J.M., Kagawa N., Waterman M.R. (1993). Expression and purification of functional human 17 alpha-hydroxylase/17,20-lyase (P450c17) in Escherichia coli. Use of this system for study of a novel form of combined 17 alpha-hydroxylase/17,20-lyase deficiency. J. Biol. Chem..

[16] Burkhard F.Z., Parween S., Udhane S.S., Flück C.E., Pandey A.V. (2017). P450 Oxidoreductase deficiency: Analysis of mutations and polymorphisms. J. Steroid Biochem. Mol. Biol..

[17] Pandey A.V., Miller W.L. (2005). Regulation of 17,20 lyase activity by cytochrome b5 and by serine phosphorylation of P450c17. J. Biol. Chem..

[18] Pandey A.V., Mellon S.H., Miller W.L. (2003). Protein phosphatase 2A and phosphoprotein SET regulate androgen production by P450c17. J. Biol. Chem..

[19] Auchus R.J., Lee T.C., Miller W.L. (1998). Cytochrome b5 augments the 17,20-lyase activity of human P450c17 without direct electron transfer. J. Biol. Chem..

[20] Zhang L.H., Rodriguez H., Ohno S., Miller W.L. (1995). Serine phosphorylation of human P450c17 increases 17,20-lyase activity: Implications for adrenarche and the polycystic ovary syndrome. Proc. Natl. Acad. Sci. USA.

[21] Idkowiak J., Randell T., Dhir V., Patel P., Shackleton C.H., Taylor N.F., Krone N., Arlt W. (2012). A missense mutation in the human cytochrome b5 gene causes 46,XY disorder of sex development due to true isolated 17,20 lyase deficiency. J. Clin. Endocrinol. Metab..

[22] DeVore N.M., Scott E.E. (2012). Structures of cytochrome P450 17A1 with prostate cancer drugs abiraterone and TOK-001. Nature.

[23] Petrunak E.M., DeVore N.M., Porubsky P.R., Scott E.E. (2014). Structures of human steroidogenic cytochrome P450 17A1 with substrates. J. Biol. Chem..

[24] Yadav R., Petrunak E.M., Estrada D.F., Scott E.E. (2017). Structural insights into the function of steroidogenic cytochrome P450 17A1. Mol. Cell Endocrinol..

[25] Auchus R.J. (2017). Steroid 17-hydroxylase and 17,20-lyase deficiencies, genetic and pharmacologic. J. Steroid Biochem. Mol. Biol..

[26] Mak P.J., Gregory M.C., Denisov I.G., Sligar S.G., Kincaid J.R. (2015). Unveiling the crucial intermediates in androgen production. Proc. Natl. Acad. Sci. USA.

[27] Geller D.H., Auchus R.J., Mendonça B.B., Miller W.L. (1997). The genetic and functional basis of isolated 17,20-lyase deficiency. Nat. Genet..

[28] Sherbet D.P., Tiosano D., Kwist K.M., Hochberg Z., Auchus R.J. (2003). CYP17 mutation E305G causes isolated 17,20-lyase deficiency by selectively altering substrate binding. J. Biol. Chem..

[29] Van Den Akker E.L., Koper J.W., Boehmer A.L., Themmen A.P., Verhoef-Post M., Timmerman M.A., Otten B.J., Drop S.L., De Jong F.H. (2002). Differential inhibition of 17alpha-hydroxylase and 17,20-lyase activities by three novel missense CYP17 mutations identified in patients with P450c17 deficiency. J. Clin. Endocrinol. Metab..

[30] Tiosano D., Knopf C., Koren I., Wudy S.A. (2008). Metabolic evidence for impaired 17alpha-hydroxylase activity in a kindred bearing the E305G mutation for isolate 17,20-lyase activity. Eur. J. Endocrinol..

[31] Parween S., Roucher-Boulez F., Flück C.E., Lienhardt-Roussie A., Mallet D., Morel Y., Pandey A.V. (2016). P450 Oxidoreductase Deficiency: Loss of Activity Caused by Protein Instability From a Novel L374H Mutation. J. Clin. Endocrinol. Metab..

[32] Flück C.E., Pandey A.V. (2017). Impact on CYP19A1 activity by mutations in NADPH cytochrome P450 oxidoreductase. J. Steroid Biochem. Mol. Biol..

[33] Pandey A.V., Sproll P. (2014). Pharmacogenomics of human P450 oxidoreductase. Front. Pharmacol..

[34] Udhane S.S., Parween S., Kagawa N., Pandey A.V. (2017). Altered CYP19A1 and CYP3A4 Activities Due to Mutations A115V, T142A, Q153R and P284L in the Human P450 Oxidoreductase. Front. Pharmacol..

[35] Geller D.H., Auchus R.J., Miller W.L. (1999). P450c17 mutations R347H and R358Q selectively disrupt 17,20-lyase activity by disrupting interactions with P450 oxidoreductase and cytochrome b5. Mol. Endocrinol..

[36] Flück C.E., Meyer-Böni M., Pandey A.V., Kempná P., Miller W.L., Schoenle E.J., Biason-Lauber A. (2011). Why boys will be boys: Two pathways of fetal testicular androgen biosynthesis are needed for male sexual differentiation. Am. J. Hum. Genet..

[37] Flück C.E., Pandey A.V. (2014). Steroidogenesis of the testis—New genes and pathways. Ann. Endocrinol..

[38] Biason-Lauber A., Miller W.L., Pandey A.V., Fluck C.E. (2013). Of marsupials and men: “Backdoor” dihydrotestosterone synthesis in male sexual differentiation. Mol. Cell Endocrinol..

[39] Audi L., Fernández-Cancio M., Carrascosa A., Andaluz P., Torán N., Piró C., Vilaró E., Vicens-Calvet E., Gussinyé M., Albisu M.A. (2010). Novel (60%) and recurrent (40%) androgen receptor gene mutations in a series of 59 patients with a 46,XY disorder of sex development. J. Clin. Endocrinol. Metab..

[40] Fernandez-Cancio M., Audí L., Andaluz P., Torán N., Piró C., Albisu M., Gussinyé M., Yeste D., Clemente M., Martínez-Mora J. (2011). SRD5A2 gene mutations and polymorphisms in Spanish 46,XY patients with a disorder of sex differentiation. Int. J. Androl..

[41] Yamazaki T., Ohno T., Sakaki T., Akiyoshi-Shibata M., Yabusaki Y., Imai T., Kominami S. (1998). Kinetic analysis of successive reactions catalyzed by bovine cytochrome p450(17alpha,lyase). Biochemistry.

[42] Soucy P., Van L.-T. (2000). Conversion of pregnenolone to DHEA by human 17α-hydroxylase/17,20-lyase (P450c17). Eur. J. Biochem..

[43] Brock B.J., Waterman M.R. (1999). Biochemical Differences between Rat and Human Cytochrome P450c17 Support the Different Steroidogenic Needs of These Two Species. Biochemistry.

[44] Pandey A.V., Kempná P., Hofer G., Mullis P.E., Flück C.E. (2007). Modulation of human CYP19A1 activity by mutant NADPH P450 oxidoreductase. Mol. Endocrinol..

[45] Flück C.E., Mullis P.E., Pandey A.V. (2010). Reduction in hepatic drug metabolizing CYP3A4 activities caused by P450 oxidoreductase mutations identified in patients with disordered steroid metabolism. Biochem. Biophys. Res. Commun..

[46] Nicolo C., Flück C.E., Mullis P.E., Pandey A.V. (2010). Restoration of mutant cytochrome P450 reductase activity by external flavin. Mol. Cell Endocrinol..

[47] Riddick D.S., Ding X., Wolf C.R., Porter T.D., Pandey A.V., Zhang Q., Gu J., Finn R.D., Ronseaux S., McLaughlin L.A. (2013). NADPH-cytochrome P450 oxidoreductase: Roles in physiology, pharmacology, and toxicology. Drug Metab. Dispos..

[48] Zalewski A., Ma N.S., Legeza B., Renthal N., Flück C.E., Pandey A.V. (2016). Vitamin D-Dependent Rickets Type 1 Caused by Mutations in CYP27B1 Affecting Protein Interactions With Adrenodoxin. J. Clin. Endocrinol. Metab..

[49] Peng H.M., Im Sa., Pearl N.M., Turcu A.F., Waskell J.R.L., Auchus R.J. (2016). Cytochrome b5 Activates the 17,20-Lyase Activity of Human Cytochrome P450 17A1 by Increasing the Coupling of NADPH Consumption to Androgen Production. Biochemistry.

[50] Yoshimoto F.K., Gonzalez E., Auchus R.J., Guengerich F.P. (2016). Mechanism of 17alpha,20-Lyase and New Hydroxylation Reactions of Human Cytochrome P450 17A1: 18O Labeling And Oxygen Surrogate Evidence For A Role Of A Perferryl Oxygen. J. Biol. Chem..

[51] Duggal R., Liu Y., Gregory M.C., Denisov I.G., Kincaid J.R., Sligar S.G. (2016). Evidence that cytochrome b5 acts as a redox donor in CYP17A1 mediated androgen synthesis. Biochem. Biophys. Res. Commun..

[52] Estrada D.F., Skinner A.L., Laurence J.S., Scott E.E. (2014). Human cytochrome P450 17A1 conformational selection: Modulation by ligand and cytochrome b5. J. Biol. Chem..

[53] Pallan P.S., Nagy L.D., Lei L., Gonzalez E., Kramlinger V.M., Azumaya C.M., Wawrzak Z., Waterman M.R., Guengerich F.P., Egli M. (2015). Structural and kinetic basis of steroid 17alpha,20-lyase activity in teleost fish cytochrome P450 17A1 and its absence in cytochrome P450 17A2. J. Biol. Chem..

[54] Ramudo Cela L., Balea-Filgueiras J., Vizoso-Hermida J.R., Martín-Herranz I. (2017). Study of cases of abiraterone discontinuation due to toxicity in pre-chemotherapy after 1 year's experience. J. Oncol. Pharm. Pract..

[55] Li Z., Alyamani M., Li J., Rogacki K., Abazeed M., Upadhyay S.K., Balk S.P., Taplin M.E., Auchus R.J., Sharifi N. (2016). Redirecting abiraterone metabolism to fine-tune prostate cancer anti-androgen therapy. Nature.

[56] Bonomo S., Hansen C.H., Petrunak E.M., Scott E.E., Styrishave B., Jørgensen F.S., Olsen L. (2016). Promising Tools in Prostate Cancer Research: Selective Non-Steroidal Cytochrome P450 17A1 Inhibitors. Sci. Rep..

[57] Sharifi N. (2015). Prostate cancer: CYP17A1 inhibitor failure-lessons for future drug development. Nat. Rev. Urol..

[58] Udhane S.S., Dick B., Hu Q., Hartmann R.W., Pandey A.V. (2016). Specificity of anti-prostate cancer CYP17A1 inhibitors on androgen biosynthesis. Biochem. Biophys. Res. Commun..

[59] Monno S., Ogawa H., Date T., Fujioka M., Miller W.L., Kobayashi M. (1993). Mutation of histidine 373 to leucine in cytochrome P450c17 causes 17 alpha-hydroxylase deficiency. J. Biol. Chem..

[60] Quattropani C., Vogt B., Odermatt A., Dick B., Frey B.M., Frey F.J. (2001). Reduced activity of 11 beta-hydroxysteroid dehydrogenase in patients with cholestasis. J. Clin. Investig..

[61] Shackleton C.H. (1993). Mass spectrometry in the diagnosis of steroid-related disorders and in hypertension research. J. Steroid Biochem. Mol. Biol..

[62] Huang N., Pandey A.V., Agrawal V., Reardon W., Lapunzina P.D., Mowat D., Jabs E.W., Van Vliet G., Sack J., Flück C.E. (2005). Diversity and function of mutations in p450 oxidoreductase in patients with Antley-Bixler syndrome and disordered steroidogenesis. Am. J. Hum. Genet..

[63] Pandey A.V., Joshi S.K., Tekwani B.L., Chauhan V.S. (1999). A colorimetric assay for heme in biological samples using 96-well plates. Anal. Biochem..

[64] Krieger E., Darden T., Nabuurs S.B., Finkelstein A., Vriend G. (2004). Making optimal use of empirical energy functions: Force-field parameterization in crystal space. Proteins.

[65] Vriend G. (1990). WHAT IF: A molecular modeling and drug design program. J. Mol. Graph..

[66] Canutescu A.A., Shelenkov A.A., Dunbrack R.L. (2003). A graph-theory algorithm for rapid protein side-chain prediction. Protein Science.

[67] Duan Y., Wu C., Chowdhury S., Lee M.C., Xiong G., Zhang W., Yang R., Cieplak P., Luo R., Lee T. (2003). A point-charge force field for molecular mechanics simulations of proteins based on condensed-phase quantum mechanical calculations. J. Comput. Chem..

[68] Jorgensen W.L., Tirado-Rives J. (2005). Potential energy functions for atomic-level simulations of water and organic and biomolecular systems. Proc. Natl. Acad. Sci. USA.

[69] Trott O., Olson A.J. (2010). AutoDock Vina: Improving the speed and accuracy of docking with a new scoring function, efficient optimization, and multithreading. J. Comput. Chem..

[70] Pettersen E.F., Goddard T.D., Huang C.C., Couch G.S., Greenblatt D.M., Meng E.C., Ferrin T.E. (2004). UCSF Chimera—A visualization system for exploratory research and analysis. J. Comput. Chem..

