# Supplementary material for: Mechanism of the Dual Activities of Human CYP17A1 and Binding to Anti-Prostate Cancer Drug Abiraterone Revealed by a Novel V366M Mutation Causing 17,20 Lyase Deficiency
Source: Pharmaceuticals (Basel). 2018 Apr 29;11(2):37. doi: 10.3390/ph11020037 (PMC6027421; doi:10.3390/ph11020037)
Supplement: Supplementary file 1 [file pharmaceuticals-11-00037-s001.pdf]

pharmaceuticals

Supplementary Materials

Mechanism of the dual activities of CYP17A1 and binding to anti-prostate cancer drug abiraterone revealed by a novel V366M mutation causing 17,20 lyase deficiency.

Mónica Fernández-Cancio[1†], Núria Camats[1,2,3†], Christa E Flück[2,3,†], Adam Zalewski[2,3,†], Bernhard Dick[4], Brigitte M Frey[4], Raquel Monné[5], Núria Torán[6], Laura Audí[1], and Amit V Pandey[2,3*]

[1] Growth and Development Research Unit, Vall d'Hebron Research Institute (VHIR), Center for Biomedical Research on Rare Diseases (CIBERER), Instituto de Salud Carlos III, Autonomous University of Barcelona, Barcelona, Spain

[2]Pediatric Endocrinology Unit, Department of Paediatrics, University Children's Hospital Bern,

[3]Department of Biomedical Research, University of Bern, Bern, Switzerland.

[4]Department of Nephrology and Hypertension, University of Bern, Bern, Switzerland.

[5]Pediatric Service, Hospital Joan XXIII, Tarragona, Spain.

[6]Pathology Department, Hospital Universitari Vall d'Hebron, CIBERER, Barcelona, Spain.

[†]Co-first authors.

* Correspondence: amit@pandeylab.org; Tel.: +41-31-632-9637

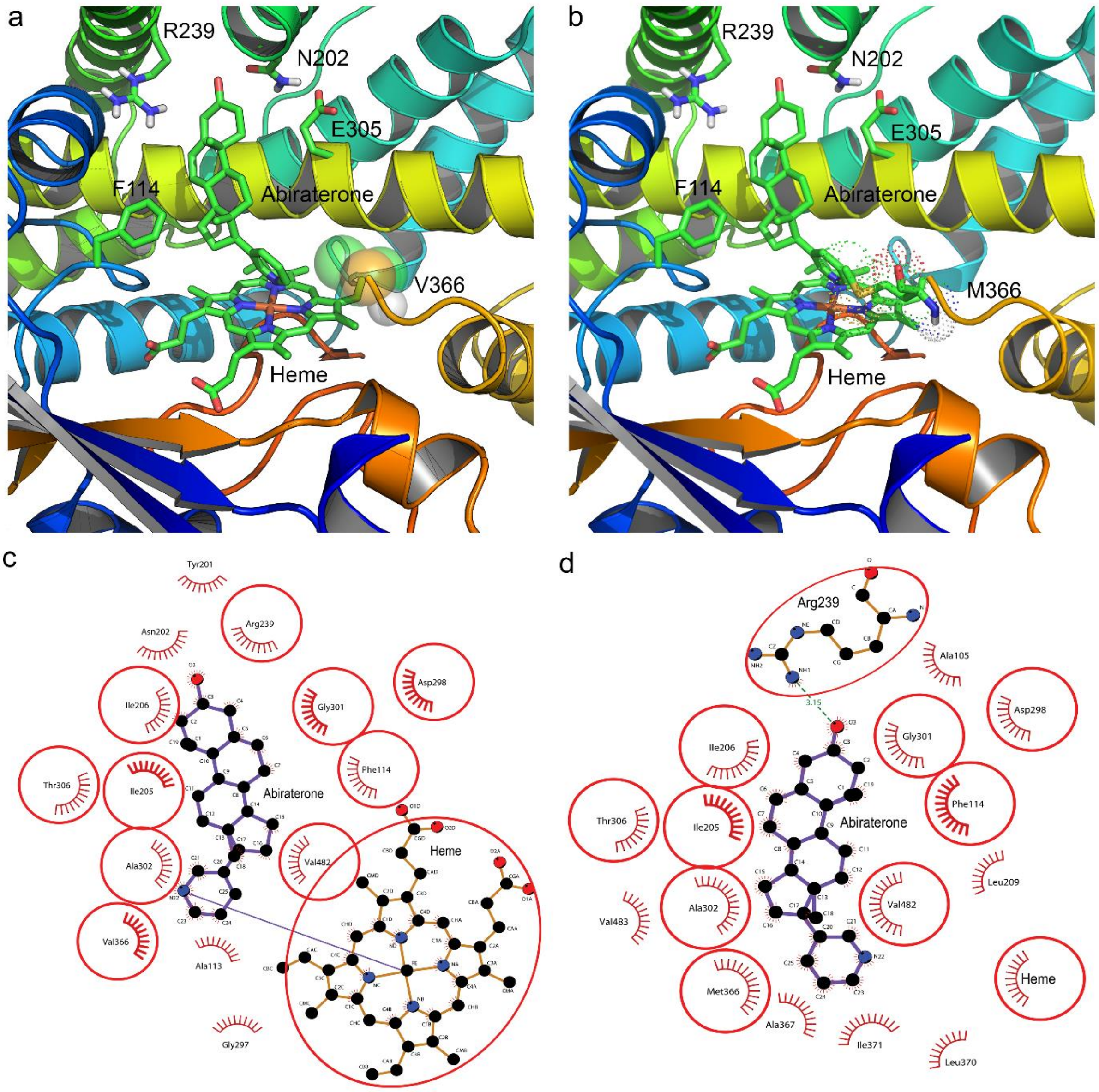

Supplementary Fig. 1: Details of abiraterone interaction with the WT and V366M variant of CYP17A1. In the WT CYP17A1 abiraterone binds by forming a nitrogen-iron co-ordination with the central heme (a and c). In the V366M mutant, the larger methionine side chain protrudes towards the heme iron and creates a steric hindrance for the binding of abiraterone (b and d). As a result, abiraterone is ineffective towards the residual 17hydroxylation reaction of the mutant enzyme. Panels a and b show the ribbons diagram of the active site of CYP17A1 while panels c and d show the ligand interactions depicted by LIGPLOT analysis.

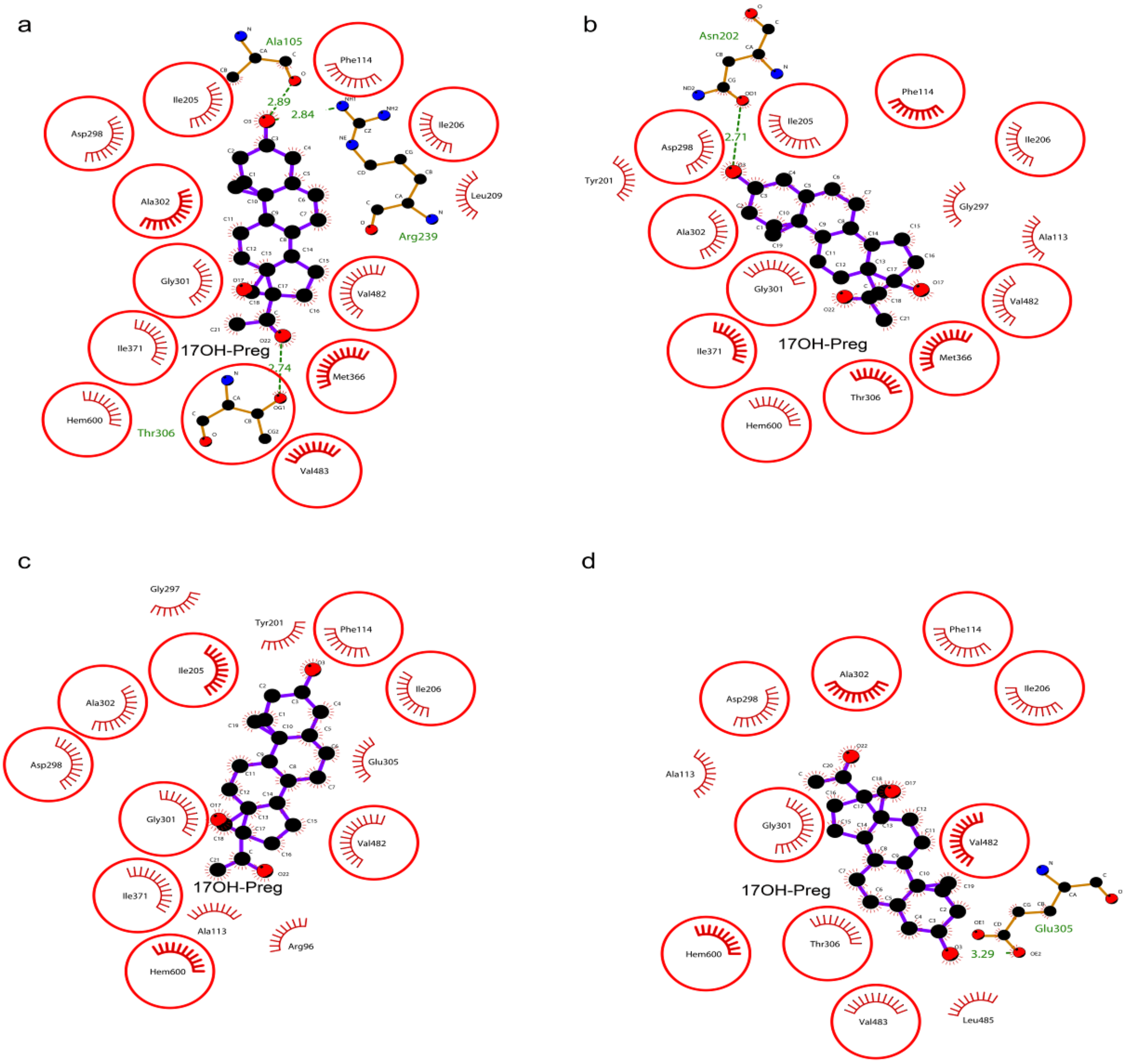

Supplementary Fig. 2: Details of 17OH-PREG interaction with the V366M variant of CYP17A1.There are differences in optimal binding poses for PREG/PROG and 17OH-PREG and while interaction with N202 seems to benefit in orienting the steroids for 17hydroxylase reaction, this increases the distance between C17 and heme iron. The multiple additional interactions observed here for 17OH-PREG and V366M mutant of CYP17A1 indicate nonoptimal binding and explain the loss of 17,20 lyase activity.
